# Supplementary material for: Gene flow relates to evolutionary divergence among populations at the range margin
Source: PeerJ. 2020 Oct 22;8:e10036. doi: 10.7717/peerj.10036 (PMC7585721; doi:10.7717/peerj.10036)
Supplement: Supplemental Information 1 — Bayesian model used to estimate traits based on sex, rearing conditions and degree of genetic isolation. Models were run for each trait (femur, wing, pronotum and genital appendages). Note that for the models for genital appendages the sex term was dropped and the models run separately for each sex. The conversion offset term was calculated for each trait and the mean difference (conversion)and its uncertainty for converting between calliper and digital measurements entered into the model as a link between the ‘true’ latent variable (z), and the observations (trait size). Data are indexed to ‘i’ at the observation level, and ‘j’ at the site level. Priors were chosen to be minimally informative to allow the data to determine their shape and range. The intercept was centred on five with a wide standard deviation (to reflect that morphological measurements should have a positive baseline value) and beta coefficients were centred on zero to not influence whether their effects were positive or negative. All priors were plotted against their posteriors to ensure the pri-or was flat in the range of the posterior (and hence, minimally informative). [file peerj-08-10036-s001.pdf]

Supplementary material for the paper

Gene flow relates to evolutionary divergence among populations at the range margin

By Peter Kaňuch, Berrit Kiehl, Anna Cassel-Lundhagen, Ane T. Laugen, Matthew Low & Åsa Berggren

Included in this supplementary material - Appendix S1

Appendix S1: General model structure

Appendix S1: Bayesian model used to estimate traits based on sex, rearing conditions & degree of genetic isolation. Models were run for each trait (femur, wing, pronotum & genital appendages). Note that for the models for genital appendages the sex term was dropped and the models run separately for each sex. The conversion offset term was calculated for each trait and the mean difference (*conversion*) and its uncertainty for converting between calliper and digital measurements entered into the model as a link between the 'true' latent variable (z), and the observations (*trait size*). Data are indexed to 'i' at the observation level, and 'j' at the site level.

Priors were chosen to be minimally informative to allow the data to determine their shape and range. The intercept was centred on 5 with a wide standard deviation (to reflect that morphological measurements should have a positive baseline value) and beta coefficients were centred on zero to not influence whether their effects were positive or negative. All priors were plotted against their posteriors to ensure the prior was flat in the range of the posterior (and hence, minimally informative).

For additional details see the BUGS code in Appendix S2.

$$\text{trait size}_i \sim Normal(z_i + conversion_i, \sigma_{obs\_i})$$

$$z_i \sim Normal(\mu_i, \sigma_{process})$$

$$\mu_i = \alpha_j + \beta_1 year_i + \beta_2 rearing_i + \beta_3 isolation_i + \beta_4 sex_i$$
$$+ \beta_5 rearing_i * isolation_i + \beta_6 rearing_i * sex_i$$
$$+ \beta_7 isolation_i * sex_i + \beta_8 rearing_i * isolation_i * sex_i$$

$$\alpha_j \sim Normal(5,30)$$

$$\beta_{1-8} \sim Normal(0,30)$$

$$\sigma_{process} \sim Uniform(0,20)$$
